# PCSK9 Expression in Vascular Smooth Muscle Cells: Role of Insulin Resistance and High Glucose

**DOI:** 10.3390/ijms26031003

**Published:** 2025-01-24

**Authors:** Cristina Barale, Giulia Tempesta, Elena Melchionda, Alessandro Morotti, Chiara Frascaroli, Alice Costanza Danzero, Saveria Femminò, Claudia Penna, Isabella Russo

**Affiliations:** 1 Department of Clinical and Biological Sciences, Turin University, 10043 Orbassano, Italy; cristina.barale@unito.it (C.B.); giulia.tempesta22@gmail.com (G.T.); elena.melchionda@unito.it (E.M.); alessandro.morotti@unito.it (A.M.); alicecostanza.danzero@unito.it (A.C.D.); saveria.femmino@unito.it (S.F.); claudia.penna@unito.it (C.P.); 2 San Luigi Gonzaga Hospital, 10043 Orbassano, Italy; c.frascaroli@sanluigi.piemonte.it

**Keywords:** proprotein convertase subtilisin/kexin type 9, vascular smooth muscle cells, insulin-resistance, high glucose, zucker rat

## Abstract

Beyond the regulation of cholesterol metabolism, a number of extrahepatic functions of proprotein convertase subtilisin/kexin type 9 (PCSK9) have been increasingly identified. The main purpose of this study was to verify whether PCSK9 expression in vascular smooth muscle cells (VSMC) is influenced by insulin resistance and high glucose (HG). In cultured rat aortic VSMC from lean insulin-sensitive Zucker rats (LZRs) and obese insulin-resistant Zucker rats (OZRs), a classical animal model of insulin resistance, we evaluated PCSK9 expression with or without the monoclonal antibodies against PCSK9 Alirocumab and Evolocumab or the synthetic PCSK9-binding peptide PEP 2-8. Effects and molecular mechanisms underlying altered PCSK9 expression were evaluated by proliferation and migration assay, reactive oxygen species (ROS) production, and involvement of PKC, NADPH-oxidase, MAPK/ERK-1/2 pathway activation. As a result, we found that, in comparison with LZR, VSMC from OZR showed basal PCSK9 overexpression mitigated by Alirocumab, Evolocumab, PEP 2-8, and the inhibitors of PKC, NADPH-oxidase, and MAPK. The finding of PCSK9 upregulation in VSMC from OZR paralleled with increased ROS production, proliferation, and migration. HG increased PCSK9 expression in VSMC from LZR, but not in OZR, via oxidative stress and with effects reduced by PCSK9 inhibitors. These findings suggest that a dysregulation of PCSK9 in VSMC could be involved in vascular damage in metabolic disorders, such as obesity and diabetes.

## 1. Introduction

Proprotein convertase subtilisin/kexin type 9 (PCSK9) is the ninth and last member of the subtilisin-like serine proteases family. PCSK9 cleaves itself, no longer functioning as a protease but acting in a non-enzymatic fashion to promote the endosomal and lysosomal degradation of receptors involved in low-density lipoprotein (LDL)-cholesterol (LDL-C) homeostasis. PCSK9 promotes LDL receptor (LDLR) degradation and recirculation to the cell surface with the consequent increase in LDLR-dependent LDL-C levels [1,2].

Even though the liver is the major source, extrahepatic organs and cells (e.g., vascular smooth muscle cells (VSMC), vascular endothelial cells, and macrophages) can release PCSK9 [3], thus contributing, in a paracrine manner, to downregulate the local LDLR expression and eventually to promote the formation of foam cells and hence atherosclerosis progression [4].

Indeed, PCSK9 can promote atherogenesis both indirectly, by increasing LDL-C plasma levels, and directly via modulation of endothelial function [5,6] and inflammatory cells such as macrophages [7] with consequent impaired vessel stability, as well as increased expression of cytokines that amplify inflammation at the atherosclerotic site.

The positive association between PCSK9 and local vessel stiffness further suggests the proatherogenic role of PCSK9, confirmed by the observation that endothelial function and arterial stiffness after short-term treatment with PCSK9 inhibitors significantly improve [8]. These findings support the concept that PCSK9 can also modulate vascular responses promoting atherogenesis in the absence of its well-established metabolic effect on LDLR modulation in hepatocytes [9,10,11]. Indeed, a number of pleiotropic extrahepatic functions of PCSK9 beyond the regulation of cholesterol metabolism have been increasingly identified [3].

Evolocumab and Alirocumab are human monoclonal antibodies neutralizing PCSK9 action with consequent reduction in LDL-C levels up to 60% [12,13,14,15]. They are indicated for the treatment of mixed dyslipidemia and primary hypercholesterolemia, particularly in patients who are ineligible for statin therapy.

Atherosclerotic plaque formation is a lipoprotein-driven disease characterized by intimal alterations of the arterial tree [16] and atherosclerotic cardiovascular disease (ASCVD) plays a major role in morbidity and mortality in diabetes and the control of risk factors is essential [17]. Type 2 diabetes mellitus (T2DM) patients, even with good glycemic control, show frequently lipid abnormalities [18]. It has been established that diabetic patients with good glycemic control and in the absence of other risk factors have a low risk of ASCVD, whereas the increasing addition of other risk factors significantly increases the risk of developing ASCVD [19]. Specifically, among the major reversible traditional risk factors in patients with diabetes, obesity (particularly visceral obesity), frequently associated with insulin resistance, and lipid abnormalities play a well-recognized role [19,20]. Thus, to reduce the risk of ASCVD in T2DM, there is an increasing need to address not only glycemic control but also the other cardiovascular (CV) risk factors.

Several studies have demonstrated that the reduction of LDL-C obtained by the administration of statins decreases ASCVD in T2DM patients [21]. However, studies also have shown that a more aggressive lipid-lowering therapy with statin and a PCSK9 inhibitor results in a greater decrease in ASCVD events than statins alone [22]. Many questions about the mechanisms by which obesity contributes to chronic conditions such as metabolic syndrome and T2DM have been clarified using animal models of genetically modified obesity. One of the most widely used models to carry out studies on obesity and metabolic syndrome is the obese Zucker rat (OZR), characterized by a homozygous leptin receptor mutation (*fa*/*fa*) that results in loss of satiety [23]. Therefore, the OZR is considered a classical animal model of insulin resistance because it presents many of the features of human insulin resistance syndrome such as obesity, chronic inflammation, and hypertension, all contributing to vascular dysfunction [24,25]. *Fa*/*fa* rats develop these metabolic abnormalities although their glucose levels are normal.

Increasing our knowledge of the molecular mechanisms involved in obesity-associated vascular diseases is a key prerequisite to identifying effective therapeutic targets to manage the clinical manifestations of obesity, T2DM, and atherosclerosis.

The main purpose of this study was to verify whether PCSK9 expression in VSMC is affected by the insulin-resistance state and high glucose exposure.

## 2. Results

### 2.1. Basal PCSK9 and Role of PCSK9 Inhibitors

**Figure 1:**
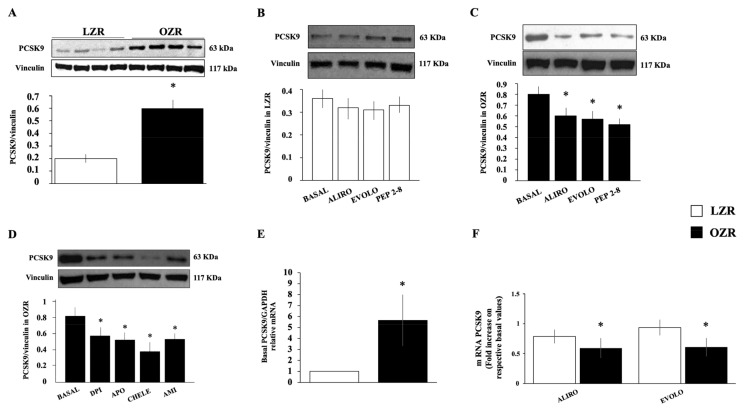
(**A**) Basal protein PCSK9 expression in vascular smooth muscle cells (VSMC) from obese insulin-resistant Zucker rat (OZR) versus VSMC from lean insulin-sensitive Zucker rat (LZR). * *p* < 0.0001 vs. LZR. (**B**,**C**) Effects of a 24-h incubation with monoclonal antibodies anti-PCSK9 Alirocumab (40 μg/mL), Evolocumab (40 μg/mL), or synthetic inhibitor PEP 2-8 (10 μmol/L) on protein PCSK9 expression in VSMC from LZR and OZR, respectively. * *p* < 0.05 vs. basal values. (**D**) Effect of a 24-h incubation with the NADPH-oxidase inhibitors diphenyleneiodonium (DPI, 10 μmol/L), apocynin (APO, 10 μmol/L), the PKC inhibitor chelerythrine (CHELE, 2.5 μmol/L), and the ROS scavenger amifostine (AMI, 50 μmol/L) on PCSK9 expression in VSMC from OZR. * *p* < 0.01 vs. basal values. (**E**,**F**) PCSK9 mRNA levels in VSMC from LZR and OZR in the absence and in the presence of PCSK9 inhibitors, respectively. * *p* < 0.05 vs. LZR.

As shown in Figure 1A, the evaluation of PCSK9 protein expression in basal condition revealed that PCSK9 levels were significantly higher in VSMC from insulin-resistant OZR (n = 12) in comparison with VSMC from lean insulin-sensitive Zucker rats (LZRs) (n = 12, *p* < 0.0001).

The preincubation for 24 h with the monoclonal antibodies Alirocumab (40 μg/mL) (Praluent^®^, Sanofi-Aventis, Bridgewater, NJ, USA) and Evolocumab (40 μg/mL) (Repatha^®^, Amgen, Thousand Oaks, CA, USA), or the synthetic inhibitor PEP 2-8 (10 μmol/L), did not significantly modify PCSK9 expression in VSMC from LZR (n = 8, ns) (Figure 1B), but reduced those from OZR (*p* < 0.005 for each inhibitor) (Figure 1C). To confirm the surprising data of basal PCSK9 upregulation in VSMC from OZR and the role of PCSK9 inhibitors, we also analyzed PCSK9 mRNA by quantitative real-time polymerase chain reaction (qRT-PCR). We found that PCSK9 mRNA levels in VSMC from OZR were upregulated compared with those from LZR (Figure 1D) (n = 5, *p* < 0.0001), and a 24 h exposure to Alirocumab (40 μg/mL), and Evolocumab (40 μg/mL) leads to a substantial reduction of mRNA in OZR (n = 4, *p* < 0.001 for both) but not in LZR (n = 4, ns) (Figure 1E). The fact that molecules neutralizing PCSK9 action can interfere on its expression induces us to hypothesize that PCSK9 positively modulates its expression.

### 2.2. PCSK9 and Oxidative Stress

To investigate a possible link between reactive oxygen species (ROS) generation and PCSK9 expression, we evaluated PCSK9 expression after inhibiting nicotinamide adenine dinucleotide phosphate hydrogen (NADPH)-oxidase, an enzyme deeply involved in ROS production in VSMC [26], and protein kinase C (PKC) which is a primary factor in NADPH-oxidase activation [27].

As shown in Figure 1E, the increased basal levels of PCSK9 in VSMC were significantly reduced by apocynin (10 μmol/L) (n = 8, *p* = 0.01) and diphenyleneiodonium (DPI) (10 μmol/L) (n = 8, *p* = 0.001). Indeed, a relevant decrease in PCSK9 protein expression was also detected in the presence of PKC inhibitor chelerythrine (2.5 μmol/L) (n = 8, *p* < 0.0001) and the antioxidant amifostine (50 μmol/L) (n = 8, *p* < 0.01).

These findings suggest the crucial role of oxidative stress in the upregulation of PCSK9 in VSMC from OZR including the activation of NADPH-oxidase and PKC pathways.

**Figure 2:**
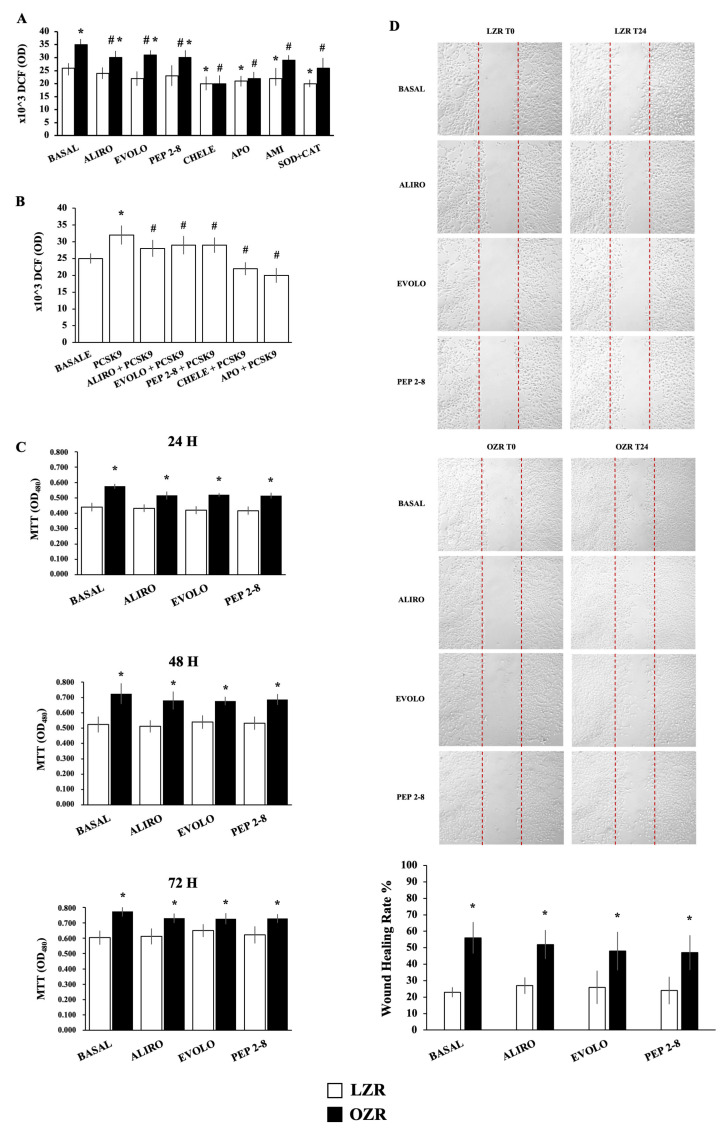
(**A**) ROS production evaluated by DCF-DA assay in VSMC from LZR and OZR after a 24-h incubation with Alirocumab (ALIRO, 40 μg/mL), Evolocumab (EVOLO, 40 μg/mL), PEP 2-8 (10 μmol/L), chelerythrine (CHELE, 2.5 μmol/L), apocynin (APO, 10 μmol/L), amifostine (AMI, 50 μmol/L), and superoxide dismutase (SOD, 300 U/mL) plus catalase (CAT, 250 U/mL). * *p* < 0.05 vs. LZR basal values. # *p* < 0.005 vs. OZR basal values. (**B**) ROS production evaluated by DCF-DA assay after a 24-h incubation with recombinant PCSK9 (5 μg/mL) in the absence and in the presence of Alirocumab (40 μg/mL), Evolocumab (40 μg/mL), PEP2-8 (10 μmol/L), chelerythrine (2.5 μmol/L), and apocynin (10 μmol/L) in VSMC from OZR. * *p* < 0.0001 vs. basal values, # *p* < 0.005 vs. rPCSK9 alone. (**C**) Proliferation assay by MTT in VSMC from LZR and OZR after a 24–48–72-h incubation with Alirocumab (40 μg/mL), Evolocumab (40 μg/mL), PEP2-8 (10 μmol/L). * *p* < 0.05 vs. LZR. (**D**) Wound healing assay at time 0 (T0) and 24 h (T24) in the absence and in the presence of Alirocumab (40 μg/mL), Evolocumab (40 μg/mL), PEP2-8 (10 μmol/L). * *p* < 0.05 vs. LZR. Data are presented as the mean ± SD.

The measurement of intracellular ROS levels, by the cell-based assays employing the fluorogenic dye, 2′,7′-dichlorodihydrofluorescein diacetate (DCF-DA) probe, revealed that basal ROS is higher in VSMC from OZR if compared with LZR (n = 18, *p* < 0.0001) (Figure 2A).

However, a significant reduction of ROS production in VSMC from OZR was observed after a 24-h treatment with chelerythrine (2.5 μmol/L) (n = 18, *p* < 0.0001), apocynin (10 μmol/L) (n = 18, *p* < 0.001), amifostine (50 μmol/L) (n = 18, *p* < 0.002), and superoxide dismutase (SOD) (300 U/mL) + catalase (250 U/mL)(n = 8, *p* < 0.0001). Interestingly, decreased ROS values in VSMC from OZR were observed after a 24-h treatment with Alirocumab (40 μg/mL), Evolocumab (40 μg/mL), or PEP 2-8 (10 μmol/L) (n = 18, *p* < 0.05 for each).

Altogether, these findings suggest that ROS modulation influences PCSK9 expression and PCSK9 inhibitors can improve redox imbalance regardless their intracellular or extracellular action.

Since PCSK9 expression paralleled ROS generation, we posited that there might be bidirectional feedback between ROS production and PCSK9 expression. For this reason, intracellular ROS levels were detected following stimulation of VSMC from LZR with recombinant PCSK9 (rPCSK9). As shown in Figure 2B, the incubation for 24 h with rPCSK9 (5 μg/mL) induced a significant increase in ROS production (n = 6, *p* < 0.01), which was reduced in the presence of PCSK9 inhibitors (n = 6, *p* < 0.05), chelerythrine (2.5 μmol/L) (n = 6, *p* < 0.005), and apocynin (10 μmol/L) (n = 6, *p* < 0.001). This suggests that PCSK9 and ROS production regulate each other’s expression.

### 2.3. Proliferation and Migration

Using 3-(4,5-dimethylthiazol-2-yl)-2,5-diphenyl tetrazolium bromide (MTT) assay, we assessed the proliferation of VSMC from LZR and OZR and the influence of PCSK9 inhibitors. We revealed that proliferation of VSMC from OZR, in comparison with LZR, was significantly higher after 24–48–72 h both in the absence and presence of Alirocumab, Evolocumab, and PEP 2-8 (n = 24, *p* = 0.01–0.0001) (Figure 2C). Therefore, VSMC show a proliferative phenotype but PCSK9 inhibitors do not seem to affect VSMC proliferation even in cells where PCSK9 is upregulated.

VSMC migration was evaluated via wound healing assay. We found that the closure rate of scratch wounds at 24 h in VSMC from OZR was much higher than in the LZR group (n = 24, *p* < 0.001). We also found that PCSK9 inhibitors did not significantly affect migration rates in both VSMC groups (n = 24, ns).

### 2.4. Phalloidin Staining and Area/Length Measurement

The phenotypic states of VSMC are essential to understanding vascular pathophysiology. VSMC generally shows contractile phenotype, but changes in contractile protein expression can lead to a phenotypic shift which is a hallmark of vascular diseases. Actually, VSMC displays extensive plasticity such that they can be stimulated to transform from a contractile state into a proliferative/migratory state.

In our study, phalloidin staining was performed to compare VSMC shape from LZR and OZR. Images from immunofluorescence confocal microscopy depict the spatial distribution of phalloidin-stained actin filaments in VSMC from insulin-sensitive LZR and insulin-resistant OZR.

**Figure 3:**
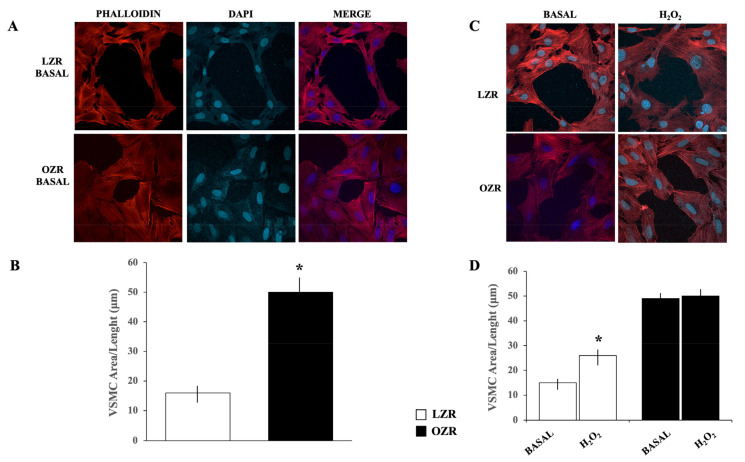
(**A**) Images from confocal laser scanning microscope captured to detect F-actin stress fiber formation, nuclei, and their merge by using phalloidin and DAPI immunofluorescence staining in aortic VSMC from LZR and OZR in basal conditions. (**B**) Calculation of cell area/length ratio in VSMC from LZR and OZR in basal conditions. * *p* < 0.0001 vs. LZR. (**C**) Effects of a 24-h incubation with H_2_O_2_ on phalloidin and DAPI immunofluorescence staining in VSMC from LZR and OZR. (**D**) Effects of a 24-h incubation with H_2_O_2_ on cell area/length ratio VSMC from LZR and OZR. * *p* < 0.05 vs. basal LZR. Data are presented as the mean ± SD.

As shown in Figure 3A, in basal conditions, VSMC from OZR, if compared with LZR, appears less fusiform and with cell size increased.

Indeed, as shown in Figure 3B, when the area/length ratio was calculated after crystal violet staining reflecting the single-VSMC morphological status, VSMC from OZR showed higher values than LZR (n = 300, *p* < 0.0001). These data suggest the tendency of OZR to change from a contractile/differentiated to a synthetic/proliferative phenotype, thus contributing to vascular remodeling.

Furthermore, as shown in Figure 3C, we also found that increasing oxidative stress by pretreating LZR VSMC with hydrogen peroxide (H_2_O_2_) (300 μmol/L for 24 h) determined morphological changes in these cells characterized by actin cytoskeleton reorganization leading to an increase in area values (Figure 3D) (n = 300, vs. basal values: *p* = 0.001). Thus, this finding demonstrates the ability of VSMC from LZR to shift to a less contractile and more synthetic phenotype if the intracellular redox balance is impaired. Therefore, an increase in oxidative stress results in the activation of signaling pathways involved in the modulation of VSMC phenotype.

### 2.5. High Glucose Effects on PCSK9 Expression

Since hyperglycemia plays a significant role in the development of T2DM complications and the condition of insulin resistance is a common feature of obese T2DM patients, we aimed to clarify whether high glucose influences VSMC PCSK9 expression.

**Figure 4:**
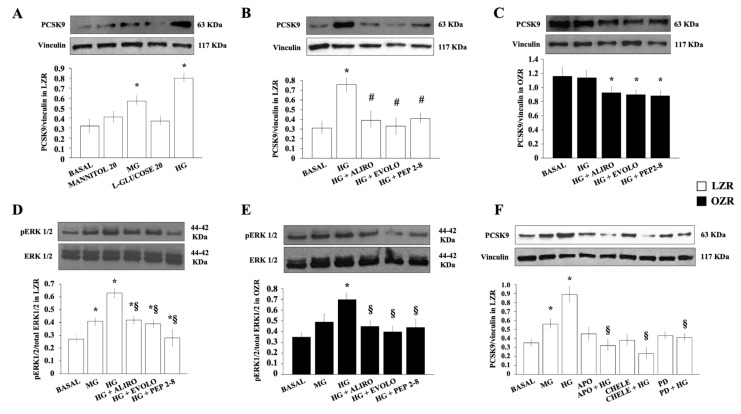
Effects of D-glucose on PCSK9 expression. (**A**) Effects of a 24 h preincubation with 15 mmol/L (middle glucose, MG), 25 mmol/L D-glucose (high glucose, HG), 20 mmol/L mannitol, and 20 mmol/L L-glucose on PCSK9 expression in aortic vascular smooth muscle cells (VSMC) from lean insulin-sensitive Zucker rat (LZR). ANOVA: *p* < 0.0001, * *p* < 0.05 vs. basal values. (**B**) Effects of the monoclonal antibody anti-PCSK9 Alirocumab (40 μg/mL), Evolocumab (40 μg/mL), synthetic PCSK9 inhibitor PEP2-8 (10 μmol/L) on the 24 hr HG-induced increase in PCSK9 expression in VSMC from LZR. * *p* < 0.0001 vs. basal values. # *p* < 0.05 vs. HG alone. (**C**) Effects of Alirocumab (40 μg/mL), Evolocumab (40 μg/mL), or PEP2-8 (10 μmol/L) on 24 hr HG-induced PCSK9 expression in VSMC from obese insulin-resistant Zucker rat (OZR). * *p* < 0.05 vs. HG alone. (**D**) Effects of a 24-h exposure to MD and HG and Alirocumab (40 μg/mL), Evolocumab (40 μg/mL), or PEP2-8 (10 μmol/L) on the HG-induced increase in pERK-1/2 in VSMC from LZR. ANOVA: *p* < 0.0001. * *p* < 0.05 vs. basal values. ^§^ *p* < 0.05 vs. HG alone. (**E**) Effects of the monoclonal antibodies anti-PCSK9 Alirocumab (40 μg/mL) and Evolocumab (40 μg/mL), and the synthetic PCSK9 inhibitor PEP2-8 (10 μmol/L) on the HG-induced increase in pERK-1/2 levels in aortic VSMC from OZR. * *p* < 0.05 vs. basal values. ^§^ *p* < 0.05 vs. HG alone. (**F**) Effects of a 24-h incubation with the PKC inhibitor chelerythrine (2.5 μmol/L), NADPH-oxidase inhibitors apocynin (10 μmol/L), and MAPK/ERK-1/2 inhibitor PD98059 (30 μmol/L) on HG-induced PCSK9 expression in VSMC from LZR. ANOVA: *p* < 0.0001. * *p* < 0.05 vs. basal values. ^§^ *p* < 0.0001 vs. HG alone. Data are presented as the mean ± SD.

We found that, in VSMC from LZR, a 24-h exposure to D-glucose (5–25 mmol/L) dose-dependently increased PCSK9 expression (n = 12, ANOVA: *p* < 0.0001) (Figure 4A). On the contrary, the PCSK9 level remained comparable to low glucose present in the basal condition (5 mM) (n = 8, *p* = ns) upon adjusting the osmolarity with mannitol or L-glucose, suggesting that hyperosmolarity associated with D-glucose 25 mmol/L (HG) had minimal effect on glucose-induced PCSK9 protein expression.

As shown in Figure 4B, the HG-induced increase in PCSK9 expression in LZR was reduced by a 30 min preincubation with Alirocumab (40 μg/mL), Evolocumab (40 μg/mL), or PEP 2-8 (10 μmol/L) (n = 12, *p* < 0.05 for all).

In VSMC from OZR, PCSK9 expression was not affected by HG (n = 12, *p* = ns) but it was reduced by the preincubation for 30 min with Alirocumab (40 μg/mL), Evolocumab (40 μg/mL), or PEP 2-8 (10 μmol/L) (n = 12, *p* < 0.05 for all) (Figure 4C).

These data induce us to hypothesize that in normal insulin-sensitive conditions, the exposure of VSMC to HG increases PCSK9 synthesis with effects, at least partially, reverted by PCSK9 inhibitors. Conversely, in the insulin-resistance state, HG is not able to further influence PCSK9 already basally upregulated, even though PCSK9 inhibitors preserve their ability to exert inhibitory effects on PCSK9 expression.

### 2.6. Effects of PCSK9 Inhibitors on the HG-Induced Activation of MAPK/ERK-1/2 Pathway

As shown in Figure 4D, in VSMC from LZR, a 24-h exposure to D-glucose dose-dependently increased phosphorylated extracellular signal-regulated kinase 1/2 (pERK)-1/2 levels (n = 10, ANOVA *p* < 0.0001) with effects reduced by the preincubation for 30 min with Alirocumab (40 μg/mL), Evolocumab (40 μg/mL), or PEP2-8 (10 μmol/L) (n = 8, *p* < 0.05 for each PCSK9 inhibitor).

In VSMC from OZR, a 24-h exposure to HG, but not to D-glucose 15 mmol/L (middle glucose, MG), increased pERK-1/2 levels which were inhibited by the preincubation for 30 min with Alirocumab (40 μg/mL), Evolocumab (40 μg/mL), or PEP 2-8 (10 μmol/L) (n = 8, *p* < 0.05 for each PCSK9 inhibitor) (Figure 4E).

These data confirm the role of HG in activating the mitogen-activated protein kinase (MAPK)/ERK-1/2 (MEK) pathway in VSMC and add knowledge on the ability of PCSK9 inhibitors to attenuate the HG-induced MAPK/ERK-1/2 activation in VSMC from both insulin-sensitive and insulin-resistant states.

### 2.7. Effects of MAPK/ERK-1/2, PKC, and NADPH-Oxidase Inhibitors on the HG-Induced Increase in PCSK9 Expression in Aortic VSMC from Insulin-Sensitive LZR

As shown in Figure 4F, in VSMC from LZR, D-glucose incubated for 24 h dose-dependently increased PCSK9 expression and the effect was inhibited by the preincubation for 30 min with apocynin (10 μmol/L), chelerythrine (2.5 μmol/L), and the MEK1 inhibitor PD098059 (30 μmol/L) (n = 8, *p* < 0.0001).

These findings clearly show the involvement of MAPK/ERK-1/2, PKC, and NADPH-oxidase pathways activation in the HG effect on PCSK9 expression in VSMC from insulin-sensitive LZR.

## 3. Discussion

In experiments with cultured aortic VSMC from LZR and OZR, we were able to identify important and new findings on the relationships between PCSK9 expression and two pathognomonic conditions underlying metabolic and vascular disorders related to T2DM: insulin resistance, and hyperglycemia. Specifically, our current research firstly demonstrated that, in comparison with VSMC from insulin-sensitive LZR, the basal expression of PCSK9 in VSMC from insulin-resistant OZR is upregulated most likely due to intracellular dysregulation leading to increased oxidative stress. Furthermore, we also demonstrated that HG increases PCSK9 expression in VSMC from insulin-sensitive LZR, but not in those from OZR, by mechanisms involving PKC, NADPH-oxidase, and MAPK/ERK-1/2 pathway activation. Interestingly, the treatment with the monoclonal antibodies against PCSK9 Alirocumab and Evolocumab was able to attenuate, on the one hand, the basal overexpression of PCSK9 in VSMC from OZR and, on the other side, the HG-induced increase in PCSK9 expression in VSMC from LZR.

In humans, PCSK9 levels significantly correlate with the classical parameters of metabolic syndrome such as obesity, serum insulin levels, fasting blood glucose, and hypertension [28], whose effects on atherosclerosis progression are also independent of LDL metabolism [29,30,31].

Circulating levels of PCSK9 predict CV risk and its increased concentrations have been found in atherosclerotic plaques [32]. The best-known function of PCSK9 is its effect on the LDLR, and circulating PCSK9 levels are associated with an increase in LDL-C. Indeed, several studies in various clinical contexts focused on PCSK9 as a marker of CV risk [32]. VSMC expresses large amounts of PCSK9 which can exert important effects on the modulation of LDLR expression in macrophages [4] and monocytes [33]. Enhanced expression of PCSK9 was found in VSMC under pro-inflammatory stimuli [33]. On the other hand, PCSK9 knock-out mice, in comparison with wild-type-ones, have VSMC with reduced proliferation rate and suppressed migratory capacity [34].

Data from Literature report that obesity upregulates PCSK9 expression which positively correlates with body mass index and high levels of PCSK9 expression are associated with disease progression [35]. We performed in vitro experiments on cultured VSMC isolated by a classical animal model of insulin resistance, the obese Zucker rat (*fa*/*fa*). This genetic overnutrition model shows a comparable representation of diet-induced human obesity conditions [36]. Surprisingly, in our in vitro studies performed on isolated cells, we found that basal expression of the protein PCSK9 was significantly higher in VSMC from OZR than in VSMC from LZR.

PCSK9 is primarily produced by the liver and increased circulating PCSK9 appears associated with metabolic disorders including insulin resistance and diabetes [37].

PCSK9 levels in obesity have been shown upregulated and VSMC can synthesize a significant amount of this protein. Although VSMC overexpression of PCSK9 may potentially contribute to increased circulating PCSK9 levels, we suppose that elevated PCSK9 release from VSMC may influence the vessel wall microenvironment and play a role in the pathological tissue function.

Furthermore, we also observed that cell treatment with the monoclonal antibodies against PCSK9 Alirocumab and Evolocumab, or the synthetic PCSK9 inhibitor PEP 2-8 is able to mitigate the increased basal PCSK9 expression in VSMC from OZR. This finding suggests that in VSMC positive feedback is triggered by PCSK9, thus justifying the ability of anti-PCSK9 antibodies to interfere with PCSK9 upregulation. Taking in mind that PCSK9 inhibitors have been developed for clinical usage of treatment of CV diseases, but their molecular mechanisms in atherosclerosis are not fully elucidated, our results suggest that the effects of Alirocumab and Evolocumab might be attributed not only to inhibition of PCSK9 action but also to their ability to attenuate its synthesis. Our data also show that PKC and NADPH-oxidase inhibitors can reduce PCSK9 overexpression in VSMC from OZR. As it is well known, PKC-mediated NADPH oxidase activation is a major source of ROS in VSMC [38]. Indeed, when ROS was measured, we found that VSMC from OZR showed impaired redox balance, thus confirming previous observations [39]. Indeed, the fact that the lowering the ROS levels significantly reduces PCSK9 expression indicates a peculiar role of oxidative stress in the activation of machinery implicated in PCSK9 synthesis. Since PCSK9 inhibition, regardless of the intracellular or extracellular action, determined a decrease in ROS production and the exposure to rPCSK9 increased ROS synthesis, we hypothesize a strong relationship between aberrant PCSK9 expression and increased oxidative stress observed in VSMC from OZR. This further highlights the role of abnormal ROS generation in the pathophysiology of CV diseases.

VSMCs, primarily quiescent and highly differentiated, are critical for maintaining vessel tone and blood pressure, thus explaining why abnormal proliferation, migration, and phenotypic switch from a contractile to a synthetic phenotype are deeply implicated in vascular injury and early atherosclerosis [40]. VSMCs are implicated in many steps of vascular diseases also due to their plasticity and ability to switch from a contractile to a proliferative state [41]. Changes in the VSMC phenotype can deeply influence VSMCs themselves and neighboring cells by the release of a wide range of bioactive molecules that can act in an autocrine and paracrine manner. Indeed, VSMC exposure to CV risk factors determines cell migration into the intima, secretion, as well as increased proliferation [42].

In our experimental conditions, in comparison with LZR, the proliferation and migration rates of VSMC from OZR were significantly higher. Furthermore, images from immunofluorescence confocal microscopy showing the spatial distribution of phalloidin-stained actin filaments revealed that VSMC from OZR shows higher values of area surface, actually reflecting a morphology shifting to a more synthetic phenotype. It is surprising that the change in morphology is evident despite removing cells from their native environment and being placed in a culture. This fact confirms the value of this genetic model of obesity which maintains in vitro peculiar characteristics despite the absence of the physiological signals that maintain and regulate the VSMC phenotype in vivo.

We also revealed the role of oxidative stress as a factor contributing to phenotype change in VSMC. In fact, the exposure of VSMC from LZR to H_2_O_2_ determined an increase in cell area surface values, indicating that oxidative stress can strongly influence VSMC phenotype.

T2DM has a great impact on the CV system and, among the intricated diabetes-related vascular complications, alterations of VSMC function are deeply involved [43,44]. It has been clearly demonstrated that in T2DM, atherosclerotic lesions are accelerated and associated with VSMC dysfunction, even though HG seems to be involved in VSMC switching from a contractile to a synthetic phenotype without direct effects on growth promotion [45]. Another main finding of the current study was the HG-induced increase in PCSK9 in VSMC from LZR, while the exposure of VSMC from OZR to HG did not significantly modify PCSK9 synthesis if compared to basal values. We also aimed to evaluate the role of PKC in the glucose-induced, oxidative stress-mediated increase in PCSK9 expression in VSMC from LZR, since one of the main mechanisms linking HG and oxidative stress is the glucose-induced activation of PKC, which in turn activates the superoxide anion generating NADPH-oxidase [46]. Among the signaling molecules involved in the HG-induced increase in PCSK9 expression, MAPK/ERK-1/2 pathway also plays a role. Actually, the inhibition of PKC by chelerythrine, NADPH-oxidase by apocynin or DPI, as well as of MAPK/ERK-1/2 by PD98058 all reduced PCSK9 expression stimulated by HG thus confirming the involvement of these pathways in HG effects. We also found that PCSK9 inhibitors similarly reduce the HG-induced activation of PKC, NADPH-oxidase, and MAPK/ERK-1/2 pathways.

The results of available epidemiological, preclinical, and clinical studies established that plasma levels of PCSK9 are significantly higher in diabetic patients than in nondiabetic individuals [47]. Despite some contradictory reports, most studies attribute to glycemic control index to a significant positive association with circulating PCSK9 levels [48]. Here, on the basis of our data, we can speculate that in this positive association, a role could be played by hyperglycemia’s effects on VSMC synthetic activity and ability to release PCSK9. In this context, the administration of PCSK9 inhibitors may also be useful to interfere with PCSK9 synthesis.

## 4. Methods

### 4.1. Chemicals

DCF-DA was from Invitrogen Molecular Probes (Paisley, Renfrewshire, UK). Recombinant PCSK9 was purchased from BPS Bioscience (San Diego, CA, USA); compounds and antibodies used for Western blots are detailed in the specific paragraphs. Unless otherwise specified, all reagents were obtained from Sigma-Aldrich (St. Louis, MO, USA).

### 4.2. Animals

Male LRZ (n = 8) and age-matched OZR (n = 8) rats, purchased from Charles River Laboratories Italy (Calco, Lecco, Italy), were fed with standard rodent chow and water ad libitum until 14 weeks old. All animals were housed in a controlled rodent facility (20~22 °C; 12 h light/dark cycle) with unlimited access to standard rodent chow and water. VSMC from LZR and OZR employed in this study were isolated from animals purchased, housed, and sacrificed for cell isolation in the year 2000, when obtaining ethical approval in Italy was not mandatory. Animal care and treatment were conducted in accordance with the institutional guidelines that are in compliance with national (Decreto Legislativo n.116, Gazzetta Ufficiale suppl 40, 18 febbraio 1992, Circolare n.8, Gazzetta Ufficiale 14 luglio 1994) and international laws and policies (EEC Council Directive 86/609, December 1987; Guide for the Care and Use of Laboratory Animals, U.S. National Research Council, 1996).

### 4.3. Cell Culture and Characterization

All the experiments were performed on cultured VSMCs obtained in our laboratory from aortas of LRZ and age-matched OZR sacrificed with CO_2_ after a 12 h fast. Aortas were removed immediately after sacrifice and VSMC isolation and characterization were made according to classical procedures, as previously reported [49]. Briefly, VSMC were cultured in minimum essential medium (MEM) (Thermo Fisher Scientific, Waltham, MA, USA) supplemented with 10% fetal bovine serum (FBS), 100 U/mL of penicillin, 100 μg/mL of streptomycin, and buffered with 10 mM of *N*-tris (hydroxymethyl) methyl-2 aminoethane-sulphonic acid (TES) and 10 mM of *N*-(2-hydroxyethyl) piperazine-N1-2-ethanesulphonic acid (HEPES). Incubation was carried out at 37 °C in a humidified incubator with an atmosphere of 95% O_2_:5% CO_2_, and the medium was replaced every 3 days. The purity of cultures was evaluated by immunofluorescence staining and by fluorescence-activated cell sorting (FACS) separation in VSMC incubated with: (i) a monoclonal antibody anti-α-smooth muscle actin, the specific marker of smooth muscle cells; (ii) monoclonal antibody anti-specific markers for endothelial cells, i.e., von Willebrand Factor (Dako, Carpinteria, CA, USA) and CD31 (Serotec, Oxford, UK), to exclude the presence of endothelial cells in the cultures; (iii) a fluorescein isothiocyanate (FITC)-conjugated secondary antibody goat anti-mouse immunoglobulins (Dako). Fixed cells were analyzed with fluorescence an LSM Zeiss confocal microscope (Carl Zeiss, Jena GmbH, Jena, Germany). Antigen expression was also analyzed with a FACS scan flow cytometer (Becton Dickinson, Milan, Italy). With FACS separation, 87–99% of the cells were positive for α-actin, and less than 1% were positive for CD31 and von Willebrand factor. For the experiments, VSMCs were used at the 7–9th passage and cultured in MEM with 10% FBS until 80% confluence was achieved. Then, MEM with 10% FBS was removed, and cells were cultured overnight in a medium with 0.1% FBS, which was changed before the experiments.

### 4.4. Protein Expression and Extent of Protein Phosphorylation by Western Blot

Proteins were extracted at the end of each experimental protocol and Western blot analyses were performed. Briefly, VSMCs were washed two times with phosphate-buffered saline (PBS) and then solubilized with boiling Laemmli buffer containing a cocktail of protease inhibitors. Then, lysates were centrifuged for 10 min at 13,000 rpm, and protein concentration was measured by the Bradford Reagent method. Cell lysates were separated by 10–12% sodium-dodecylsulphate-polyacrylamide gel electrophoresis (SDS-PAGE) and transferred to polyvinylidene fluoride membrane (PVDF) (Immunological Sciences, Rome, Italy). Membranes were saturated with a 5% milk solution and tween tris buffered saline, and then incubated overnight at 4 °C with primary antibody. Then, blots were incubated with a peroxidase-conjugate secondary antibody for 45 min. After washing, proteins were detected with Enhanced ChemiLuminescence (ECL Westar Supernova, Cyanagen Srl, Bologna, Italy). The dilution of each antibody was as follows: rabbit anti-PCSK9 (1:200) (Calbiochem, EMD Chemicals San Diego, CA, USA), rabbit anti-vinculin (1:1000) (Santa Cruz Biotechnology, Dallas, TX, USA), mouse anti-ERK-1/2 (1:1000) (Santa Cruz Biotechnology), rabbit anti-pERK-1/2 (1:1000) (Immunological Sciences). Membranes were then incubated with secondary antibodies conjugated with horseradish peroxidase and as above mentioned, visualized using chemiluminescence. A 45 min incubation was performed for secondary goat anti-rabbit (1:10,000) (Cell Signaling, Danvers, MA, USA) in PBS containing 0.1% Tween-20, or goat anti-mouse (1:1000) (Jackson ImmunoResearch, West Grove, PA, USA) in PBS containing 0.1% Tween-20.

### 4.5. mRNA PCSK9 Expression by RT-qPCR

Following the manufacturer’s instructions, total RNA was isolated from VSMC from LZR and OZR using an RNeasy Mini Kit (Qiagen, Hilden, Germany). RNA purity was assessed by spectrometry at an absorbance of 260 nm and was deemed valid if the A260/A280 ratio fell within the range of 1.8 to 2.0. Total RNA was reverse transcribed into complementary DNA using the High-Capacity cDNA Reverse Transcription Kit (Thermo Fisher Scientific), following the manufacturer’s recommendations. Quantification of PCSK9 and glyceraldehyde-3-phosphate dehydrogenase (GAPDH) mRNA was performed by RT-qPCR using the SensiFAST™ SYBR^®^ No-ROX Kit (Meridian Bioscience, Cincinnati, OH, USA) on a CFX96™ System (BioRad, Hercules, CA, USA). Samples were analyzed in duplicate, with 200 ng of initial RNA loaded per well. All results were normalized against GAPDH, and the relative ratio was calculated using the comparative CT method (ΔΔCT value). Primers, used at 400 nM final concentration, have the following sequences: PCSK9 Forward primer (5′-3′): GCTTCAGCGGCTTGTTCCT; PCSK9 Reverse primer (5′-3′): TGCTCCTCCACTCTCCACATAA; GAPDH Forward primer (5′-3′): TGGCCTCCAAGGAGTAAGAAAC; GAPDH Reverse primer (5′-3′): GGCCTCTCTCTTGCTCTCAGTATC. The relative ratio was calculated using the comparative CT method (ΔΔCT value). All results were normalized against GAPDH.

### 4.6. ROS Detection by DCF-DA Assay

ROS were measured by using the DCF-DA assay, more specifically for the detection of H_2_O_2_. This assay was performed by using DCF-DA, a diacetylated fluorescence probe that diffuses into the cells, where intracellular esterases cleave the acetyl groups, and are oxidized by H_2_O_2_ to the highly fluorescent DCF [50]. In our experiment, VSMCs were cultured in 96-multiwell plates (1 × 10^5^ mL^−1^) and incubated with MEM with 0.1% FBS. After treatment, the medium was aspirated, and cells were washed with PBS containing 1 mM CaCl_2_ and 1 mM MgCl_2_ (PBS^+^) and incubated in the dark for 30 min at 37 °C in the presence of 10 µM of DCF-DA. After that, cells were washed with PBS^+^, and the emitted DCF fluorescence was collected and measured using a plate fluorometer, Tecan Infinite 200 microplate reader (Tecan Group Ltd., Männedorf, Switzerland) fitted with 490 nm excitation and 520 nm emission filters. Each assay was carried out with six replicates and the fluorescence measure for each treatment was the average value of at least three independent experiments.

### 4.7. Cell Proliferation Assay by MTT Assay

MTT assay was used to measure cell proliferation as indicated by the mitochondrial reduction of MTT to the purple formazan. VSMC (~1000 cells/well) were plated in 96-well culture plates. After incubating the cells overnight with 10% FBS MEM to allow them to adhere, the cells were left for 24 h with 0.1% FBS, then maintained in MEM with 10% FBS, and treated with the appropriate stimuli. After 24–48–72 h, the media in each microwell was replaced with 100 μL of a (1:5) dilution of MTT stock solution in MEM. After incubation at 37 °C and 5% CO_2_ for ~2 h, the MTT solution was replaced with 100 μL of dimethyl sulfoxide (DMSO) and incubated at 37 °C and 5% CO_2_ for 15 min. The absorbance of the solubilized formazan was then read at 570 nm (reference wavelength 690 nm) using a plate reader ChroMate 4300 (Awareness Technology Inc., Palm City, FL, USA). All experiments were performed at least three times.

### 4.8. Wound Healing Assay

VSMC were cultivated to reach at least 80% confluence after being seeded in 6-well plates at a density of 2.0 × 10^5^ cells per well. After removing the culture medium, artificial wounds were made in each well using a 100 µL sterile pipette tip. The tip was inserted vertically into all the wells, to ensure that the width of each scratch was almost identical for each well. The width of each scratch was measured as the baseline value. PBS was used to wash the scratched cells off, and they were added to a serum-free medium. Samples and photographs were taken at 0 and 24 h and the width of the scratch was observed with an inverted microscope AE2000 (MOTIC, Barcelona, Spain), original magnification 4×, and measured by Wound Healing Size Tool, an ImageJ/Fiji software (v. 2.14.0) plugin. The closure percentage of the scratch was calculated as below: wound healing rate (%) = (A_0_ − A_t_)/A_0_ × 100%. A_0_ represented the initial wound area and A_t_ represented the remaining wound area when the measurement was done.

### 4.9. Phalloidin Staining

The morphological changes and F-actin stress fiber formation were detected on VSMC by phalloidin immunofluorescence staining. When VSMC reached a confluence of approximately 70%, they were seeded onto a 6-well cell culture plate containing approximately 10,000 cells on a glass slide in each well. After treatment, cells were washed three times with PBS (5 min each time). Following this, they were fixed with 4% paraformaldehyde at room temperature for 30 min. The slides were rinsed three times with PBS (5 min each time) and permeabilized with 0.5% TritonX-100 at room temperature for 20 min. After washing with PBS, cells were blocked with 5% BSA for 30 min, then stained with 5 U/mL phalloidin (tetramethyl-rhodamine B isothiocyanate) (Sigma-Aldrich) in a dark chamber at 37 °C for 1 h. Nuclei were counterstained with 4′-6-diamidino-2-phenylindole (DAPI) (Sigma-Aldrich). Cells were examined under a confocal laser scanning microscope BD FACS Lyric (Becton Dickinson).

### 4.10. Measurement of Cell Area Surface and Perimeter by Crystal Violet Staining Assay

Cell size was determined using crystal violet staining. Approximately 8.000 cells/well of VSMC were plated in 6-well culture plates and allowed to adhere overnight, then the 10% FBS medium was replaced with 0.1% FBS overnight. Cells were washed with PBS and stained with 1% crystal violet solution and washed 6 times with PBS after 10 min to remove the surplus stain. The samples were photographed with a microscope AE2000 (MOTIC, Barcelona, Spain) (magnification ×10) and cell size was determined using Motic Images Plus 3.0 software. At least 50 cells were measured in each well and the experiments were repeated six times.

### 4.11. Statistical Analysis

Data are expressed as mean ± SD. Each experiment was replicated at least three times, with n indicating the number of biological replicates. Significance was evaluated, when appropriate, by unpaired Student’s *t*-test and by one-way ANOVA followed by Bonferroni’s analysis. A *p* < 0.05 was considered significant.

## 5. Conclusions

Collectively, the findings of this study suggest that (i) in the proatherogenic condition of obesity associated with insulin resistance, the upregulation of PCSK9 in VSMC could play a role; (ii) in the absence of insulin resistance, HG may play a role in the increased levels of PCSK9 in diabetes also for its effects on VSMC, thus adding a further mechanism by which hyperglycemia, in lean subjects, can promote vascular atherosclerotic process; (iii) the beneficial effects of PCSK9 inhibitors on atherosclerosis could be also due to their effects on VSMC both in insulin-sensitive, for the attenuation of the HG-induced increase in PCSK9 expression, and insulin-resistant state, for the attenuation of basal PCSK9 expression.

## Data Availability

The original contributions presented in this study are included in the article. Further inquiries can be directed to the corresponding author.

## References

[1] Horton J.D., Cohen J.C., Hobbs H.H. (2007). Molecular Biology of PCSK9: Its Role in LDL Metabolism. Trends Biochem. Sci..

[2] Barale C., Melchionda E., Morotti A., Russo I. (2021). PCSK9 Biology and Its Role in Atherothrombosis. Int. J. Mol. Sci..

[3] Cariou B., Si-Tayeb K., Le May C. (2015). Role of PCSK9 beyond Liver Involvement. Curr. Opin. Lipidol..

[4] Ferri N., Tibolla G., Pirillo A., Cipollone F., Mezzetti A., Pacia S., Corsini A., Catapano A.L. (2012). Proprotein Convertase Subtilisin Kexin Type 9 (PCSK9) Secreted by Cultured Smooth Muscle Cells Reduces Macrophages LDLR Levels. Atherosclerosis.

[5] Li J., Liang X., Wang Y., Xu Z., Li G. (2017). Investigation of Highly Expressed PCSK9 in Atherosclerotic Plaques and Ox-LDL-Induced Endothelial Cell Apoptosis. Mol. Med. Rep..

[6] Ding Z., Liu S., Wang X., Deng X., Fan Y., Sun C., Wang Y., Mehta J.L. (2015). Hemodynamic Shear Stress via ROS Modulates PCSK9 Expression in Human Vascular Endothelial and Smooth Muscle Cells and along the Mouse Aorta. Antioxid. Redox Signal.

[7] Ricci C., Ruscica M., Camera M., Rossetti L., Macchi C., Colciago A., Zanotti I., Lupo M.G., Adorni M.P., Cicero A.F.G. (2018). PCSK9 Induces a Pro-Inflammatory Response in Macrophages. Sci. Rep..

[8] Cicero A.F.G., Toth P.P., Fogacci F., Virdis A., Borghi C. (2019). Improvement in Arterial Stiffness after Short-Term Treatment with PCSK9 Inhibitors. Nutr. Metab. Cardiovasc. Dis..

[9] Leander K., Mälarstig A., Van’t Hooft F.M., Hyde C., Hellénius M.-L., Troutt J.S., Konrad R.J., Öhrvik J., Hamsten A., de Faire U. (2016). Circulating Proprotein Convertase Subtilisin/Kexin Type 9 (PCSK9) Predicts Future Risk of Cardiovascular Events Independently of Established Risk Factors. Circulation.

[10] Xie W., Liu J., Wang W., Wang M., Qi Y., Zhao F., Sun J., Liu J., Li Y., Zhao D. (2016). Association between Plasma PCSK9 Levels and 10-Year Progression of Carotid Atherosclerosis beyond LDL-C: A Cohort Study. Int. J. Cardiol..

[11] Denis M., Marcinkiewicz J., Zaid A., Gauthier D., Poirier S., Lazure C., Seidah N.G., Prat A. (2012). Gene Inactivation of Proprotein Convertase Subtilisin/Kexin Type 9 Reduces Atherosclerosis in Mice. Circulation.

[12] Roth E.M. (2019). Alirocumab for Low-Density Lipoprotein Cholesterol Lowering. Future Cardiol..

[13] Kasichayanula S., Grover A., Emery M.G., Gibbs M.A., Somaratne R., Wasserman S.M., Gibbs J.P. (2018). Clinical Pharmacokinetics and Pharmacodynamics of Evolocumab, a PCSK9 Inhibitor. Clin. Pharmacokinet..

[14] Sabatine M.S., Giugliano R.P., Keech A.C., Honarpour N., Wiviott S.D., Murphy S.A., Kuder J.F., Wang H., Liu T., Wasserman S.M. (2017). Evolocumab and Clinical Outcomes in Patients with Cardiovascular Disease. N. E. J. Med..

[15] Schwartz G.G., Steg P.G., Szarek M., Bhatt D.L., Bittner V.A., Diaz R., Edelberg J.M., Goodman S.G., Hanotin C., Harrington R.A. (2018). Alirocumab and Cardiovascular Outcomes after Acute Coronary Syndrome. N. E. J. Med..

[16] Bentzon J.F., Otsuka F., Virmani R., Falk E. (2014). Mechanisms of Plaque Formation and Rupture. Circ. Res..

[17] Low Wang C.C., Hess C.N., Hiatt W.R., Goldfine A.B. (2016). Clinical Update: Cardiovascular Disease in Diabetes Mellitus: Atherosclerotic Cardiovascular Disease and Heart Failure in Type 2 Diabetes Mellitus—Mechanisms, Management, and Clinical Considerations. Circulation.

[18] Ginsberg H.N., MacCallum P.R. (2009). The Obesity, Metabolic Syndrome, and Type 2 Diabetes Mellitus Pandemic: Part I. Increased Cardiovascular Disease Risk and the Importance of Atherogenic Dyslipidemia in Persons with the Metabolic Syndrome and Type 2 Diabetes Mellitus. J. Cardiometab Syndr..

[19] Chakraborty S., Verma A., Garg R., Singh J., Verma H. (2023). Cardiometabolic Risk Factors Associated with Type 2 Diabetes Mellitus: A Mechanistic Insight. Clin. Med. Insights Endocrinol. Diabetes.

[20] Martín-Timón I., Sevillano-Collantes C., Segura-Galindo A., Del Cañizo-Gómez F.J. (2014). Type 2 Diabetes and Cardiovascular Disease: Have All Risk Factors the Same Strength?. World J. Diabetes.

[21] Jialal I., Singh G. (2019). Management of Diabetic Dyslipidemia: An Update. World J. Diabetes.

[22] Jamadade P., Nupur N., Maharana K.C., Singh S. (2024). Therapeutic Monoclonal Antibodies for Metabolic Disorders: Major Advancements and Future Perspectives. Curr. Atheroscler. Rep..

[23] Kasiske B.L., O’Donnell M.P., Keane W.F. (1992). The Zucker Rat Model of Obesity, Insulin Resistance, Hyperlipidemia, and Renal Injury. Hypertension.

[24] Anfossi G., Russo I., Doronzo G., Trovati M. (2009). Contribution of Insulin Resistance to Vascular Dysfunction. Arch. Physiol. Biochem..

[25] Anfossi G., Russo I., Doronzo G., Pomero A., Trovati M. (2010). Adipocytokines in Atherothrombosis: Focus on Platelets and Vascular Smooth Muscle Cells. Mediators Inflamm..

[26] Kim S.G., Sung J.Y., Kang Y.J., Choi H.C. (2023). Fisetin Alleviates Cellular Senescence through PTEN Mediated Inhibition of PKCδ-NOX1 Pathway in Vascular Smooth Muscle Cells. Arch. Gerontol. Geriatr..

[27] Rastogi R., Geng X., Li F., Ding Y. (2016). NOX Activation by Subunit Interaction and Underlying Mechanisms in Disease. Front. Cell Neurosci..

[28] Ruscica M., Macchi C., Giuliani A., Rizzuto A.S., Ramini D., Sbriscia M., Carugo S., Bonfigli A.R., Corsini A., Olivieri F. (2023). Circulating PCSK9 as a Prognostic Biomarker of Cardiovascular Events in Individuals with Type 2 Diabetes: Evidence from a 16.8-Year Follow-up Study. Cardiovasc. Diabetol..

[29] Shin D., Kim S., Lee H., Lee H.-C., Lee J., Park H.-W., Fukai M., Choi E., Choi S., Koo B.-J. (2024). PCSK9 Stimulates Syk, PKCδ, and NF-κB, Leading to Atherosclerosis Progression Independently of LDL Receptor. Nat. Commun..

[30] Dutka M., Zimmer K., Ćwiertnia M., Ilczak T., Bobiński R. (2024). The Role of PCSK9 in Heart Failure and Other Cardiovascular Diseases-Mechanisms of Action beyond Its Effect on LDL Cholesterol. Heart Fail. Rev..

[31] Patriki D., Saravi S.S.S., Camici G.G., Liberale L., Beer J.H. (2022). PCSK 9: A Link Between Inflammation and Atherosclerosis. Curr. Med. Chem..

[32] Vlachopoulos C., Terentes-Printzios D., Georgiopoulos G., Skoumas I., Koutagiar I., Ioakeimidis N., Stefanadis C., Tousoulis D. (2016). Prediction of Cardiovascular Events with Levels of Proprotein Convertase Subtilisin/Kexin Type 9: A Systematic Review and Meta-Analysis. Atherosclerosis.

[33] Grune J., Meyborg H., Bezhaeva T., Kappert K., Hillmeister P., Kintscher U., Pieske B., Stawowy P. (2017). PCSK9 Regulates the Chemokine Receptor CCR2 on Monocytes. Biochem. Biophys. Res. Commun..

[34] Ferri N., Marchianò S., Tibolla G., Baetta R., Dhyani A., Ruscica M., Uboldi P., Catapano A.L., Corsini A. (2016). PCSK9 Knock-out Mice Are Protected from Neointimal Formation in Response to Perivascular Carotid Collar Placement. Atherosclerosis.

[35] Mbikay M., Chrétien M. (2022). The Biological Relevance of PCSK9: When Less Is Better…. Biochem. Cell Biol..

[36] Zhang Y., Proenca R., Maffei M., Barone M., Leopold L., Friedman J.M. (1994). Positional Cloning of the Mouse Obese Gene and Its Human Homologue. Nature.

[37] Cui C.-J., Li S., Li J.-J. (2015). PCSK9 and Its Modulation. Clin. Chim. Acta.

[38] Lassègue B., Clempus R.E. (2003). Vascular NAD(P)H Oxidases: Specific Features, Expression, and Regulation. Am. J. Physiol. Regul. Integr. Comp. Physiol..

[39] Russo I., Viretto M., Doronzo G., Barale C., Mattiello L., Anfossi G., Trovati M. (2014). A Short-Term Incubation with High Glucose Impairs VASP Phosphorylation at Serine 239 in Response to the Nitric Oxide/cGMP Pathway in Vascular Smooth Muscle Cells: Role of Oxidative Stress. Biomed. Res. Int..

[40] Chen R., McVey D.G., Shen D., Huang X., Ye S. (2023). Phenotypic Switching of Vascular Smooth Muscle Cells in Atherosclerosis. J. Am. Heart Assoc..

[41] Basatemur G.L., Jørgensen H.F., Clarke M.C.H., Bennett M.R., Mallat Z. (2019). Vascular Smooth Muscle Cells in Atherosclerosis. Nat. Rev. Cardiol..

[42] Raines E.W., Ross R. (1993). Smooth Muscle Cells and the Pathogenesis of the Lesions of Atherosclerosis. Br. Heart J..

[43] Meigs J.B., Larson M.G., D’Agostino R.B., Levy D., Clouse M.E., Nathan D.M., Wilson P.W.F., O’Donnell C.J. (2002). Coronary Artery Calcification in Type 2 Diabetes and Insulin Resistance: The Framingham Offspring Study. Diabetes Care.

[44] Chen Z., Li X., Sun X., Xiao S., Chen T., Ren L., Liu N. (2024). STING1-Accelerated Vascular Smooth Muscle Cell Senescence-Associated Vascular Calcification in Diabetes Is Ameliorated by Oleoylethanolamide via Improved Mitochondrial DNA Oxidative Damage. Free Radic. Biol. Med..

[45] Yang J., Gourley G.R., Gilbertsen A., Chen C., Wang L., Smith K., Namenwirth M., Yang L. (2024). High Glucose Levels Promote Switch to Synthetic Vascular Smooth Muscle Cells via Lactate/GPR81. Cells.

[46] Inoguchi T., Sonta T., Tsubouchi H., Etoh T., Kakimoto M., Sonoda N., Sato N., Sekiguchi N., Kobayashi K., Sumimoto H. (2003). Protein Kinase C-Dependent Increase in Reactive Oxygen Species (ROS) Production in Vascular Tissues of Diabetes: Role of Vascular NAD(P)H Oxidase. J. Am. Soc. Nephrol..

[47] Lakoski S.G., Lagace T.A., Cohen J.C., Horton J.D., Hobbs H.H. (2009). Genetic and Metabolic Determinants of Plasma PCSK9 Levels. J. Clin. Endocrinol. Metab..

[48] Momtazi A.A., Banach M., Pirro M., Stein E.A., Sahebkar A. (2017). PCSK9 and Diabetes: Is There a Link?. Drug Discov. Today.

[49] Doronzo G., Russo I., Mattiello L., Anfossi G., Bosia A., Trovati M. (2004). Insulin Activates Vascular Endothelial Growth Factor in Vascular Smooth Muscle Cells: Influence of Nitric Oxide and of Insulin Resistance. Eur. J. Clin. Invest..

[50] LeBel C.P., Ischiropoulos H., Bondy S.C. (1992). Evaluation of the Probe 2′,7′-Dichlorofluorescin as an Indicator of Reactive Oxygen Species Formation and Oxidative Stress. Chem. Res. Toxicol..

